# Role of Donepezil in Autism: Its Conduciveness in Psychopharmacotherapy

**DOI:** 10.1155/2011/563204

**Published:** 2011-11-17

**Authors:** Rohit Kant Srivastava, Medhavi Agarwal, Ashish Pundhir

**Affiliations:** Department of Psychiatry, Rohilkhand Medical College, Bareilly, Uttar Pradesh 243006, India

## Abstract

A woman consulted psychiatric Out-Patient Department (OPD) for her 5-year and 2-month-old son presenting with typical autistic symptoms like social, behavioural, and communicational ineptitudeness. Subsequent treatment with Donepezil resulted in marked improvement in the aforementioned symptomatology. Recent studies in autistic child have shown diminished acetylcholine and nicotinic receptor activity, thus an acetylcholinergic enhancer, Donepezil, likely accounts for improvement in autistic symptoms. Evidently, the case report consolidates Donepezil role as a potentially useful agent in the treatment of cognitive and behavioural symptoms observed in this disorder.

## 1. Background

Autism spectrum disorders (ASDs) are characterized by qualitative impairments in reciprocal social interaction and communication and by the presence of restricted repetitive behaviour [1]. ASD occurs more often in boys than girls, with a 4 : 1 male to female ratio [2].

Most recent reviews tend to estimate a prevalence of 1-2 per 1000 for autism and close to 6 per 1000 for autistic spectrum disorder [3]; because of inadequate data, these numbers may underestimate autistic spectrum disorders true prevalence [4].

Autism affects information processing in the brain by altering how nerve cells and their synapses connect and organize; how this occurs is not well understood [5]; the search for a single anatomical or petrochemical deficit in autism has been unsuccessful to date. However, recent research by Perry and her colleagues [6] examined basal forebrain and cerebral cortex of adult autistic brains and described frontal lobe region versus controls [6]. These findings support that pharmacotherapy may ameliorate acetylcholinergic receptors by increasing the concentration of available acetylcholine. There are neuropathological and neurochemical abnormalities of the cholinergic pathway in autism. Thus we observed the role of Donepezil in autism.

## 2. Case Details

A 5-year and 2-month-old boy was reported to psychiatric Out-Patient Department (OPD) along with his mother for psychiatric evaluation who told the consultant that her boy has impairment in social interaction and in communication and inadequate appreciation of socioeconomic cues, as shown by impairment in use of nonverbal behavior such as poor eye to eye contact, not showing facial expression and gestures to regulate social interaction. He had lack of social or emotional reciprocity. He had poor peer relationship. He also had impairment in communication, marked impairment in the ability to initiate or sustain conversation with others. On the basis of ICD-10 he is diagnosed to have childhood autism. He also has hyperactivity, aggression, and self-injury (e.g., wrist biting). On application of VSMS his I.Q. was found to be 90. On application of childhood autism rating scale (CARS) his score was 50. Both parents were explained about the nature of illness. They were also explained about the educational and behavioral interventions and structural training programme needed for treatment of autism; a facilitated communication technique was started by a clinical psychologist. Along with behavior therapy he was given risperidone 0.5 mg. 1 HS for hyperactivity, and response was awaited for 8 weeks but there was no improvement so Donepezil as an add-on treatment in a dose of 5 mg. 1 HS was administered. Patient started having meaningful improvement within next 6 weeks on reapplication of CARS, his score became 38.

He got major improvements in communication. He started having eye to eye contact, started having better touch-smell-taste response, better verbal communication, decreased hyperactivity, and better emotional response.

## 3. Discussion

Substantial benefit in reducing inattention and hyperactivity is seen with atypical antipsychotics such as risperidone [7]. But on the contrary, no improvement for hyperactivity in this case was witnessed.

Hardan and Handen [8] on conducting retrospective pilot study to determine the effectiveness of Donepezil in the treatment of children and adolescents with autism observed decrease in the irritability and hyperactivity subscale but no changes in the inappropriate speech, lethargy, and stereotypes subscales were noted.

On the contrary, the result of our observation found Donepezil an acetylcholinesterase inhibitor, when used in autism improves the symptoms of autism especially in receptive and expressive language as well as overall general autistic features.

After 6 weeks on Donepezil, changes in expressive and receptive speech were

1. apparent by parents,
2. significant improvement was shown by CARS.

The significance of changes in our measurements of vocabulary and autistic behavior was further supported by parental reports of verbal expressions that appeared to become more laden with emotional content; increased fluency and reduced dysarthria were also observed when compared to the often nonrhythmic, monotone speech prior to treatment. Greater ease in nonverbal imitation was also reported.

Moderate benefit is derived from methylphenidate, atomoxetine, some anticonvulsant medications guanfacine, and Donepezil [7]. The adjective “moderate” should be alternated with “drastic”, as Donepezil use has miraculous impact on subsidence of social, behavioural, and communicational ineptitudeness.

Handen et al. placebo controlled trial of Donepezil in 34 children and adolescents with ASD concluded short-term treatment with Donepezil may have limited impact on cognitive functioning in ASD [9].

 The most common adverse event was the concomitant increase in mood swings and lability [10]. Dunn et al. on studying adverse effect associated with Donepezil witnessed that the commonest adverse events were nausea, diarrhea, malaise, dizziness, and insomnia [11]. However, in our case parents did not report any significant noticeable adverse effect after adding Donepezil.

Chez et al. trial of Donepezil Hydrochloride in pediatric autistic spectrum patients suggested that pharmacological stimulation of acetyl cholinergic receptors can improve receptive and expressive language as well as overall general autistic features [10].

In this case, we also witnessed improvement in receptive and expressive language after adding Donepezil.

The quest for a reliable, standardized, normalized rating instrument to assess clinical efficacy in clinical drug trials in the field of autism remains a current subject of inquiry [12].

Despite these caveats Donepezil was found to be effective in autism. Clinical observation supported alterations in exploratory behaviours with the use of Donepezil.
